# Prevalent *ALMS1* Pathogenic Variants in Spanish Alström Patients

**DOI:** 10.3390/genes12020282

**Published:** 2021-02-16

**Authors:** Brais Bea-Mascato, Carlos Solarat, Irene Perea-Romero, Teresa Jaijo, Fiona Blanco-Kelly, José M. Millán, Carmen Ayuso, Diana Valverde

**Affiliations:** 1 CINBIO, Universidad de Vigo, 36310 Vigo, Spain; bbea@uvigo.es (B.B.-M.); Carlos.lopez.solarat@uvigo.es (C.S.); 2 Grupo de Investigación en Enfermedades Raras y Medicina Pediátrica, Instituto de Investigación Sanitaria Galicia Sur (IIS Galicia Sur), SERGAS-UVIGO, 36310 Vigo, Spain; 3 Centro de Investigación Biomédica en Red en Enfermedades Raras (CIBERER), ISCIII, 28029 Madrid, Spain; irene.perea@quironsalud.es (I.P.-R.); jaijo_ter@gva.es (T.J.); fblancok@quironsalud.es (F.B.-K.); jose_millan@iislafe.es (J.M.M.); CAyuso@fjd.es (C.A.); 4 Departamento de Genética Clínica, Instituto de Investigación Sanitaria Hospital Universitario Fundación Jiménez Díaz, (IIS-FJD, UAM), 28040 Madrid, Spain; 5 Unidad de Genética, Hospital Universitario y Politécnico La Fe. Biomedicina Molecular Celular y Genómica, Instituto Investigación Sanitaria La Fe, 46026 Valencia, Spain

**Keywords:** ciliopathies, Alström syndrome, metabolic disease, novel mutations, founder effect

## Abstract

Alström syndrome (ALMS) is an ultrarare disease with an estimated prevalence lower than 1 in 1,000,000. It is associated with disease-causing mutations in the Alström syndrome 1 (*ALMS1*) gene, which codifies for a structural protein of the basal body and centrosomes. The symptomatology involves nystagmus, type 2 diabetes mellitus (T2D), obesity, dilated cardiomyopathy (DCM), neurodegenerative disorders and multiorgan fibrosis. We refined the clinical and genetic diagnosis data of 12 patients from 11 families, all of them from Spain. We also studied the allelic frequency of the different variants present in this cohort and performed a haplotype analysis for the most prevalent allele. The genetic analysis revealed 2 novel homozygous variants located in the exon 8, p.(Glu929Ter) and p.(His1808GlufsTer20) in 2 unrelated patients. These 2 novel variants were classified as pathogenic after an *in silico* experiment (computer analysis). On the other hand, 2 alleles were detected at a high frequency in our cohort: p.(Tyr1714Ter) (25%) and p.(Ser3872TyrfsTer19) (16.7%). The segregation analysis showed that the pathogenic variant p.(Tyr1714Ter) in 3 families is linked to a rare missense polymorphism, p.(Asn1787Asp). In conclusion, 2 novel pathological mutations have been discovered in homozygosis, as well as a probable founder effect in 3 unrelated families.

## 1. Introduction

Alström Syndrome (ALMS; OMIM #203800) is an ultrarare recessive disorder, with an estimated prevalence lower than 1 in 1,000,000 in European-descent populations. As in the case of other rare syndromes, consanguineous and/or geographically isolated populations have higher frequency values [1,2,3]. About 1000 cases have been described worldwide for this pathology, of which 13 have been diagnosed in Spain [4,5,6,7].

ALMS is a pleiotropic and multisystemic disorder characterized by a high inter- and intrafamilial variability, regarding the phenotype displayed, the age of onset and the severity of symptoms [4,5]. The cardinal features include childhood obesity, insulin resistance, cone-rod retinal dystrophy, sensorineural hearing loss, type 2 diabetes mellitus (T2D), hypertriglyceridemia and dilated cardiomyopathy (DCM) [8]. Other secondary features include seizures, hyporeflexia or multiorgan fibrosis that develops from adolescence onwards. This latter is very variable and can affect the liver, kidneys, lungs and gonads [8]. The first clinical feature, visual dysfunction (photophobia and nystagmus), usually develops between a few weeks after birth and the first year of life [5]. The remaining signs evolve slowly during childhood and adolescence, although the most severe features can be detected before the first decade [4].

The clinical diagnosis of ALMS is based on the presence of primary and secondary features, considering the age of onset throughout the development [9].

ALMS is a monogenic disorder caused by pathogenic variants in the *ALMS1* gene (MIM #606844), which represents an unusual phenomenon among ciliopathies, normally described with high genetic heterogeneity. *ALMS1* is located on chromosome 2 (region 2p13.1) and consists of 224 kilobases (kb) containing 23 coding exons [10,11]. Several splicing isoforms have been reported, which could produce different protein isoforms with specific functions [10,12,13,14]. 

To date, over 298 pathogenic variants have been involved in ALMS development, of which 96% are nonsense or frameshift changes (insertions and deletions) that could originate truncated, nonfunctional proteins [4,15]. Most of the deleterious variants are clustered in exons 8 (6.1 kb), 10 (1.9 kb) and 16 (1.2 kb), which are considered mutational hotspots as they comprise 85–97% of the total mutational load for *ALMS1* in the different cohorts [4,15]. Hence, the direct sequencing of these 3 exons represents the standard strategy when ALMS is suspected. However, the progressive implementation of high-throughput sequencing (HTS) techniques, such as whole-exome sequencing (WES) and targeted gene panels, is replacing the classical approach to ALMS molecular diagnosis [16,17,18].

The vast majority of changes in *ALMS1* have been described once, most cases worldwide are compound heterozygotes, and several groups of patients have shown a founder effect, like the Acadian, English or Turkish population [1,3,19]. The knowledge of the mutational load in this gene could be interesting for understanding the genotype-phenotype correlation and the molecular basis of this disorder.

## 2. Materials and Methods

### 2.1. Cohort Presentation

This study included 12 patients from 11 unrelated families clinically diagnosed with ALMS (Patients 4 and 5 are siblings). Here, we reported the genetic characterization of 5 males and 7 females (Table 1) of Caucasian ethnicity.

The clinical history for 11 of the 12 patients was obtained through collaboration with medical doctors and the National Association of Alström syndrome Spain. The main clinical characteristics are described in Table 2.

Most of the families were molecularly described elsewhere [4], and, as part of the Spanish ciliopathy cohort, they have been studied clinically and molecularly by our group [6,7]. Families GBB-28 and UG-26225 have been described for the first time in this study, and their molecular characterization was deposited in ClinVar.

### 2.2. DNA Extraction and Sanger Sequencing

DNA was extracted from peripheral blood from participants (Patients 1, 3, 6, 7 and 8) and available family members. We used the Flexigene DNA kit 250 (Qiagen, Hilden, Germany), following the manufacturer’s protocol. 

After DNA extraction, we analyzed the exonic DNA of *ALMS1*. We amplified the DNA by polymerase chain reaction (PCR) in an MJ MiniTM Gradient Thermal Cycler (Bio-Rad, Hercules, CA, USA) with the primers described by Collin et al. [10] (Table S2). PCR reactions were performed using 100 ng of genomic DNA, 1 μL at 10 μM of each primer and 12.5 µL Supreme NZYTaq II 2x Green Master Mix (Nzytech, Lisbon, Portugal) in a final volume of 25 µL per sample. The amplification program applied to samples was as follows: initial denaturing at 95 °C for 5 min, followed by 35 cycles of 94 °C for 30 s, 52–66 °C for 30 s, and 72 °C for 30 s and a final extension step at 72 °C for 10 min [6]. Then, the PCR products were resolved and stained on a 2% agarose gel with 0.05% ethidium bromide.

After that, 4.5 µL of each PCR was purified using ExoSAP (Thermo-Fisher, Waltham, CA, USA) in a final volume of 6 µL, incubating the reaction for 15 min at 37 °C and 15 min at 80 °C. The products were sequenced directly using the BigDye® Terminator v1.3 Cycle Sequencing Kit (Life Technologies, Foster City, CA, USA) in a 10 μL reaction. The program was as follows: initial denaturing at 98 °C for 3 min, followed by 25 cycles of 96 °C for 10 s, 50 °C for 5 s and 60 °C for 4 min. The sequencing products were precipitated and dried using MgCl_2_, ethanol at 4 °C and Microfuge 18® (Beckman-Coulter™, Krefeld, Germany) [6]. The final product was resolved in an ABI PRISM 3130 (Life Technologies, Foster City, CA, USA) genetic analyzer.

Finally, all sequences were visualized with the BioEdit 7.2, and the reference sequences ENST00000613296.6/ENSP00000482968.1 were used for the nucleotide and amino acid numbering of pathogenic variants. 

### 2.3. Relative Allele Frequency Calculation

The calculation of the relative allele frequency (P) was performed using the number of alleles with a specific change (i) divided by the total number of alleles in our cohort (*N* = 24). Then, the result was multiplied by 100 to obtain a percentage.

P = i/N * 100

### 2.4. In Silico Analysis of Variants

To predict the mutational effect, the novel pathogenic *ALMS1* variants were analyzed with the following most used software: PolyPhen2 (p. (Glu929Ter) mutation was excluded from this analysis because the software does not analyze STOP mutations, considering them to always be pathogenic) [21], SIFT [22], MutPred-LOF [23] and PROVEAN [24]. The score provided by these software were used to classify the variants following the American College of Medical Genetics and Genomics (ACMG) guidelines [25].

## 3. Results

### 3.1. Patients Characteristics

All of the 12 patients, (5 males and 7 females) from the 11 families have a positive molecular diagnosis that is biallelic according to a recessive model.

In all cases, we established 2 pathogenic variants. Families carrying the same variant did not have any kindred relationship between them and came from different Spanish locations across the country.

We detected 11 different pathogenic variants (Table 1), 6 patients from the families, GBB-45, GBB-46, RP-793, RP-1232, GBB-28 and UG-26225, being homozygous. Regarding pathogenic variants, 1 is located in exon 5 [20], 7 are located in exon 8 [4,6,7], 1 in exon 9 [4], 1 in exon 16 [19] and 1 in exon 17 [4] of the *ALMS1* gene, and all of them lead to a stop codon. The variant p.(Tyr1714Ter) in exon 8 has a high frequency in our pool of patients, at 25%, appearing 6 times in 5 patients (1 homozygous and 4 compound heterozygous). The pathogenic variant p.(Ser3872TyrfsTer19) has been detected 4 times in 3 patients (1 homozygous and 2 compound heterozygous), rising to a 16.7% frequency. Most of the variants detected as being pathogenic were not shown in the gnomAD and ClinVar databases (Table S1), as no population information was available. 

Some of the mutations have been uploaded into the LOVD database from the REWBA project by the labs where the molecular analysis was performed. For the data that was found, the pathogenic variant p.(Tyr1714Ter) has been described 5 times [4,15], including 3 of our samples. The pathogenic variants p.(Ser3872TyrfsTer19) and p.(Val3596GlufsTer4) have been described 3 times [4,9], a heterozygous sample for p.(Ser3872TyrfsTer19) that has been reported is 1 of our patients.

All patients included in this study present cone-rod dystrophy and/or nystagmus. Obesity (BMI > 95%), overweight (BMI > 85%), insulin resistance or T2D were present in 91% of the cases. DCM was found in 6 of 11 patients (54.5%). Hearing loss was reported in 8 of 11 patients (73%) (Table 2). 

The second group of symptoms with a low incidence was reported too: hepatic dysfunction (55%), renal failure (18%), short stature (45%), thyroid disorders (55%) and hypogonadism/irregular menses (36%) (Table 2).

### 3.2. Novel ALMS1 pathogenic variants

#### 3.2.1. Patient 1 (Family GBB-28)

Patient 1 is a young girl (11 years old) from consanguineous parents. The main symptoms of this patient are nystagmus, photophobia, and rod and cone dystrophy with decreased visual acuity; morbid obesity, DCM and bronchospasm. No further Alström spectrum symptoms have been reported to date (Table 2).

**Figure 1:**
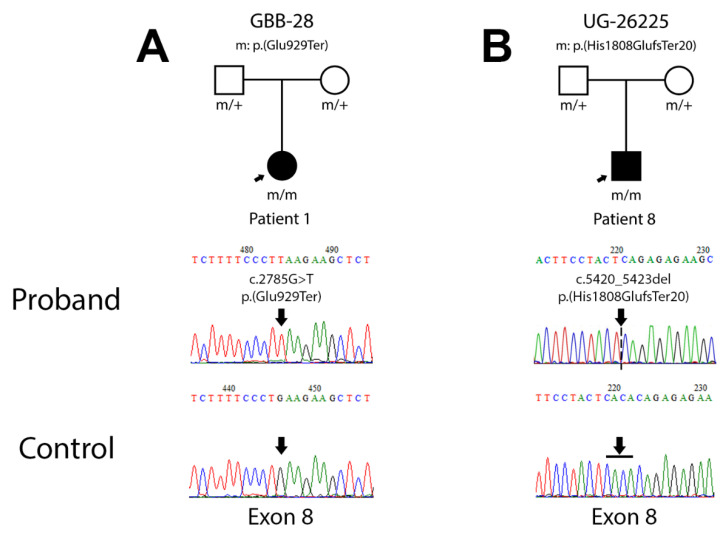
Pedigree chart and electropherogram for the patients carrying novel mutations. (**A**) Pedigree chart for family GBB-28, carrier of mutation c.2785C>T; p.(Glu929Ter) and electropherogram of the proband sequence compared to the control. (**B**) Pedigree chart for family UG-26225, carrier of mutation c.5420_5423del; p.(His1808GlufsTer20) and electropherogram of the proband sequence compared to the control.

A novel homozygous amino acid change on exon 8, c.2785G>T leading to p.(Glu929Ter), was detected by Sanger sequencing in this patient (Figure 1A). The *in silico* prediction of pathogenicity through different bioinformatic tools resulted in deleterious variant scores in MutPred-LOF (0.432), SIFT (0) and PROVEAN (−3.376). According to the ACMG, this variant should be classified as pathogenic (PVS1, very strong evidence of pathogenicity) (Table 3).

#### 3.2.2. Patient 8 (Family UG-26225)

Patient 8 is a young boy (3 years old) from consanguineous parents. The main symptoms of this patient are horizontal nystagmus within a few months of birth, DCM of birth, obesity and hypothyroidism. He has a normal size and sexual development according to his age (Table 2).

A novel homozygous amino acid change on exon 8 of *ALMS1*, c.5420_5423del leading to p.(His1808GlufsTer20), was detected by Sanger sequencing in this patient (Figure 1B). In this case, the *in silico* analysis shows deleterious scores in PolyPhen2 (0.852), MutPred-LOF (0.422), SIFT (0.05) and PROVEAN (−2.720). Following the criteria of ACMG, this variant should also be classified as pathogenic (PVS1, very strong evidence of pathogenicity) (Table 3).

### 3.3. Relative Allele Frequencies

**Figure 2:**
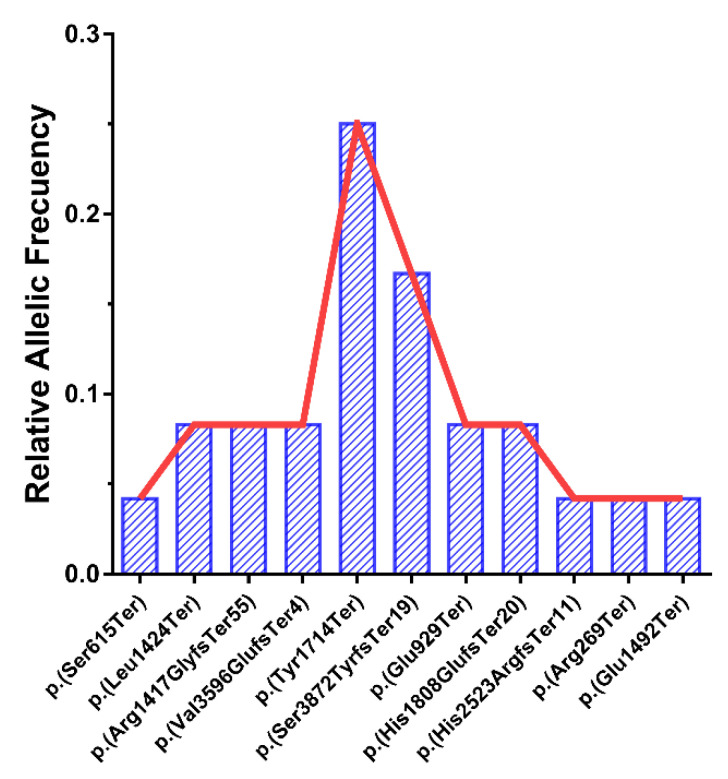
Different relative allele frequencies for the pathogenic variants detected in the cohort, expressed as a fraction of total alleles (*N* = 24).

We detected 2 specific alleles with a high frequency in *ALMS1*: p.(Tyr1714Ter) and p.(Ser3872TyrfsTer19), with only 1 family for each of these pathogenic variants being homozygous. The relative frequencies of these alleles in the cohort were 0.25 (25%) and 0.167 (16.7%), respectively (Figure 2). 

### 3.4. Segregation Study

**Figure 3:**
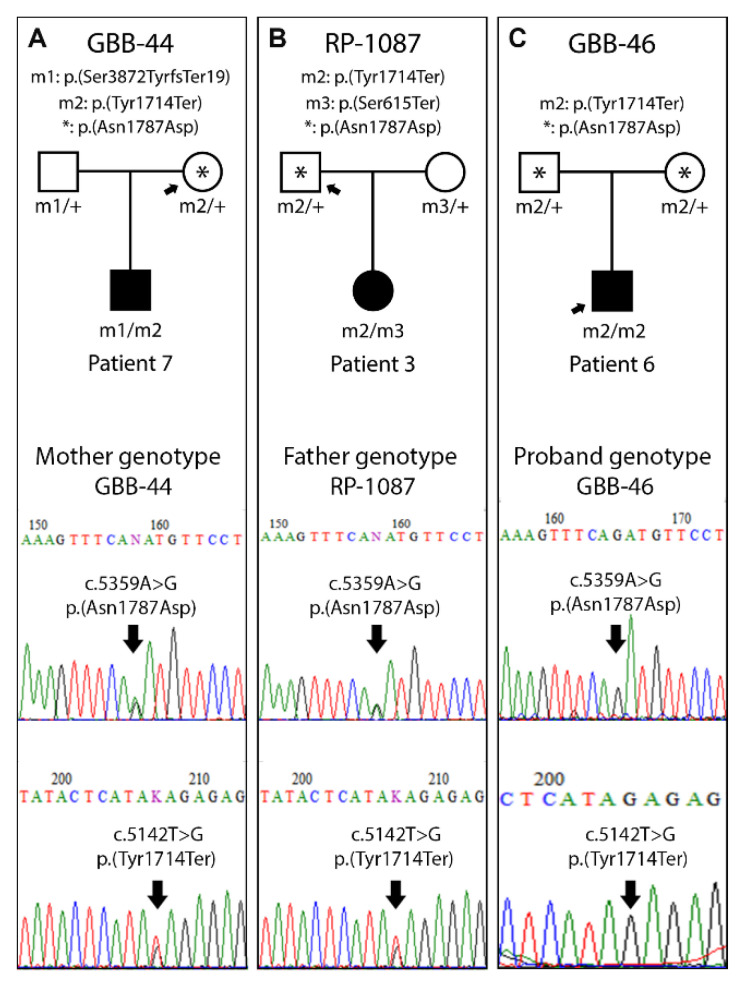
Single nucleotide polymorphism (SNP) segregation study in 3 families. (**A**) The mother´s genotype from family GBB-44, carrier of p.(Asn1787Asp) and p.(Tyr1714Ter) in heterozygosis. (**B**) The father´s genotype from the RP-1087 family, carrier of p.(Asn1787Asp) and p.(Tyr1714Ter) in heterozygosis. (**C**) The genotype of the GBB-46 proband with p.(Asn1787Asp) and p.(Tyr1714Ter) in homozygosis. *: p.(Asn1787Asp) (c.5359A>G; rs45608038).

In patients carrying the p.(Tyr1714Ter) pathogenic variant, we detected a single nucleotide polymorphism (SNP) with a low frequency (0.017) in the European population. This SNP, p.(Asn1787Asp) (c.5359A>G; rs45608038), is located at exon 8 of *ALMS1* (Table 4). Thus, we evaluated whether the allele p.(Asn1787Asp) segregated with the pathogenic variant p.(Tyr1714Ter) in 3 families, and we concluded that it was linked to the latter (Figure 3).

### 3.5. Haplogroup Classification

To complete the analysis and determine if this was a common allele, we included the SNPs described by Scheinfeldt [26] to classify the haplogroup of these patients. The 3 patients carrying p.(Tyr1714Ter) show the ancestral haplotype described for *ALMS1* (Table 4).

## 4. Discussion

Alström Syndrome is a complex disease that affects multiple organs and induces a metabolic disorder. Its huge heterogenic interpatient symptomatology and its low incidence in population worldwide makes it very difficult to perform any phenotype–genotype correlation.

In our cohort, we analyzed 12 patients from 11 families with ALMS pathogenic variants. Most of them have been previously clinically and molecularly characterized [4,6,7,19], but patient 1 (GBB-28) and patient 8 (UG-26225) have been described in this study for the first time. These 2 patients are carriers for novel *ALMS1* pathogenic variants in homozygosity. The analysis of the open reading frame (ORF) sequence showed the generation of a premature stop codon, resulting in a truncated protein in both cases. For patient 1 (GBB-28), the amino acid change affects the glutamate located in position 929 generating a stop codon (TAA). Regarding patient 8 (UG-26225), the microdeletion of 4 pb (CACA) changes the histidine in position 1808 to glutamate and generates a frameshift mutation that leads to a stop codon (TGA), 20 amino acids downstream. Until now, approximately 298 pathogenic or likely pathogenic variants have been described in *ALMS1*. A great percentage of cases harbour private mutations. Here, we are expanding this mutational spectrum with 2 novel *ALMS1* mutations.

Moreover, 2 highly prevalent pathogenic variants were detected within our cohort. Both pathogenic variants, p.(Ser3872TyrfsTer19) and p.(Tyr1714Ter), located in exons 17 and 8, respectively, generate a premature stop codon resulting in a truncated protein. In this point 1 of these pathogenic variants, p.(Tyr1714Ter), cosegregates with a low-frequency SNP, p.(Asn1787Asp) (c.5359A>G; rs45608038), in the 3 analyzed families (Figure 2), which allows us to establish a potential common origin of this allele in these Spanish patients. Furthermore, based on the haplogroups described for *ALMS1*, this haplotype is grouped with the ancestral [26], which has been detected in the south of Europe (France, Spain and Portugal) and has a high presence in the African continent. This fact could be explained as an introduction of this ALMSallele in the Iberian Peninsula from the African continent.

In this study, no genotype–phenotype correlation for the pathogenic variants p.(Tyr1714Ter) and p.(Ser3872TyrfsTer19) was detected. Even in siblings with the same genotype (p.(Tyr1714Ter)/p.(Leu1424Ter); patients 4 and 5, family GAS-37), the phenotype was not the same. In this case, patient 4 reported DCM, but his brother did not.

Due to the complexity of ALMS, the effect of only one gene does not seem to be enough to completely explain the heterogeneous symptoms seen in these patients. The prevalence of symptoms like DCM, hepatic dysfunction or hearing loss could be conditioned by other agents like common mutational load or epigenetic regulation, whose study would be interesting.

ALMS is a very rare disease that shares clinical features with other ciliopathic syndromes, so a clinical diagnosis is quite difficult in some cases due to this phenotypic overlap, highlighting the Bardet–Biedl syndrome (in presenting a multiorgan pathology) and Usher syndrome (in combining retinitis pigmentosa with hearing loss). The difficulty in achieving a diagnosis and the lack of a global point of view leads to the fact that some patients are still underdiagnosed and seek medical attendance when the symptoms are exacerbated. Given that the sample size is one of the main barriers to establishing a genotype–phenotype correlation in rare diseases, it would be interesting to establish an international registry of patients that reflects the causal mutations of each patient accompanied by their standardized symptoms. In this respect, an international effort is underway to enrol these patients into national and international associations that provide updated information to patients and put them in touch with clinicians and investigations [27] that facilitate future observational studies and clinical trials.

## 5. Conclusions

In all, we described 2 novel ALMS pathogenic variants in the exon 8 of the *ALMS1* gene that leads to a truncated protein. These 2 variants were reported in homozygosity in nonrelated patients, which showed an Alström syndrome clinical spectrum according to age. Furthermore, we detected 2 prevalent *ALMS1* pathogenic variants, p.(Tyr1714Ter) and p.(Ser3872TyrfsTer19), in our Spanish cohort. The pathogenic variant p.(Tyr1714Ter) cosegregates with a benign missense variant, p.(Asn1787Asp). Finally, 3 families with the p.(Tyr1714Ter) pathogenic variant shared the ancestral haplotype for *ALMS1* that is predominant in the African continent, which could have arisen by a founder effect.

## Figures and Tables

**Table 1:** Summary of the genotype of patients, their author reference, and the family and patient codes. The reference sequence for *ALMS1* (ENST00000613296.5/ ENSP00000482968.1) was used.

				Allele 1			Allele 2		
Patient	Family	Reference	*ALMS1* Pathogenic Variant 1 c.DNA	Exon	ALMS1 Pathogenic Variant 1 Protein	*ALMS1* Pathogenic Variant 2 c.DNA	Exon	ALMS1 Pathogenic Variant 2 Protein	Genotype Status
1	GBB-28	This study	c.2785G>T	8	p.(Glu929Ter)	c.2785G>T	8	p.(Glu929Ter)	Homozygous
2	RP-1232	[7]	c.4249del	8	p.(Arg1417GlyfsTer55)	c.4249del	8	p.(Arg1417GlyfsTer55)	Homozygous
3	RP-1087	[6]	c.5142T>G	8	p.(Tyr1714Ter)	c.1844C>G	8	p.(Ser615Ter)	Heterozygous
4	GAS-37	Allele 1 [6]; Allele 2 [4]	c.5142T>G	8	p.(Tyr1714Ter)	c.4271T>G	8	p.(Leu1424Ter)	Heterozygous
5	GAS-37	Allele 1 [6]; Allele 2 [4]	c.5142T>G	8	p.(Tyr1714Ter)	c.4271T>G	8	p.(Leu1424Ter)	Heterozygous
6	GBB-46	Allele 1 [6]; Allele 2 [4]	c.5142T>G	8	p.(Tyr1714Ter)	c.5142T>G	8	p.(Tyr1714Ter)	Homozygous
7	GBB-44	Allele 1 [6]; Allele 2 [4]	c.5142T>G	8	p.(Tyr1714Ter)	c.11615_11616del	17	p.(Ser3872TyrfsTer19)	Heterozygous
8	UG-26225	This study	c.5420_5423del	8	p.(His1808GlufsTer20)	c.5420_5423del	8	p.(His1808GlufsTer20)	Homozygous
9	RP-2186	[4]	c.7568_7569del	9	p.(His2523ArgfsTer11)	c.4474G>T	8	p.(Glu1492Ter)	Heterozygous
10	RP-793	[19]	c.10787_10788del	16	p.(Val3596GlufsTer4)	c.10787_10788del	16	p.(Val3596GlufsTer4)	Homozygous
11	GBB-45	[4]	c.11615_11616del	17	p.(Ser3872TyrfsTer19)	c.11615_11616del	17	p.(Ser3872TyrfsTer19)	Homozygous
12	RP-2177	Allele1 [4]; Allele 2 [20]	c.11615_11616del	17	p.(Ser3872TyrfsTer19)	c.805C>T	5	p.(Arg269Ter)	Heterozygous

**Table 2:** Phenotype summary based on the diagnostic criteria for Alström syndrome according to Marshall et al. (2007) [9] for 11 of the 12 cases. The clinical history of patient GBB-45 was not available. x: presence of symptom. -: absence of symptom.

Patient	Family	Sex	Age (Years)	Vision (History of Nystagmus in Infancy/Childhood, Legal Blindness, Cone and Rod Dystrophy by ERG)	Obesity and/or Insulin Resistance and/or T2D	History of DCM/CHF	Hearing Loss	Hepatic Dysfunction	Renal Failure	Short Stature	Males: Hypogonadism; Females: Irregular Menses and/or Hyperandrogenism	Thyroid Disorders	Predicted Protein Change
1	GBB-28	F	13	x	x	x	-	-	-	-	-	-	p.(Glu929Ter)/p.(Glu929Ter)
2	RP-1232	F	27	x	x	-	x	-	x	-	x	x	p.(Arg1417GlyfsTer55)/p.(Arg1417GlyfsTer55)
3	RP-1087	F	42	x	x	-	x	x	x	x	x	-	p.(Tyr1714Ter)/p.(Ser615Ter)
4	GAS-37	F	21	x	x	x	x	x	-	x	x	x	p.(Tyr1714Ter)/p.(Leu1424Ter)
5	GAS-37	M	26	x	x	-	x	x	-	x	x	x	p.(Tyr1714Ter)/p.(Leu1424Ter)
6	GBB-46	M	23	x	x	-	x	x	-	x	-	x	p.(Tyr1714Ter)/p.(Tyr1714Ter)
7	GBB-44	M	18	x	x	x	x	x	-	-	-	-	p.(Tyr1714Ter)/p.(Ser3872TyrfsTer19)
8	UG-26225	M	3	x	x	x	-	-	-	-	-	x	p.(His1808GlufsTer20)/p.(His1808GlufsTer20)
9	RP-2186	M	9	x	x	x	-	-	-	-	-	-	p.(His2523ArgfsTer11)/p.(Glu1492Ter)
10	RP-793	F	11	x	x	-	x	x	-	x	-	x	p.(Val3596GlufsTer4)/p.(Val3596GlufsTer4)
12	RP-2177	F	49	x	-	x	x	-	-	-	-	-	p.(Ser3872TyrfsTer19)/p.(Arg269Ter)

ERG: Electroretinogram; T2D: Type 2 Diabetes Mellitus; DCM: Dilated Cardiomyopathy; CHF: Congestive Heart Failure.

**Table 3:** Scores obtaining from the *in silico* analysis of pathogenicity for the 2 novel mutations p.(Glu929Ter) and p.(His1808GlufsTer20) and their classification according to American College of Medical Genetics and Genomics (ACMG) guidelines. The 4 programs used for this analysis were: PolyPhen2, MultiPred-LOF, SIFT and PROVEAN.

Patient	Family	*ALMS1* Pathogenic Variant c.DNA	Exon	*ALMS1* Pathogenic Variant Protein	PolyPhen2	MutPred-LOF	SIFT	PROVEAN	ACMG
1	GBB-28	c.2785G>T	8	p.(Glu929Ter)	-	0.432	0	−3.376	Pathogenic
8	UG-26225	c.5420_5423del	8	p.(His1808GlufsTer20)	0.852	0.422	0,05	−2.720	Pathogenic

**Table 4:** Haplogroup classification of Alström patients with the pathogenic variant p.(Tyr1714Ter). Analyzed SNP, predicted protein change, exon in which they are found, genotype of the study individuals and shared common allele.

Predicted Protein Change	SNPs	Exon	GBB-44	RP-1087	GBB-46	Common Allele
p.(Phe730=)	rs7598901	8	T/T	T/T	T/T	T
p.(Gly1415Val)	rs6546837	8	G/G	C/G	G/G	G
p.(Ile1876Val)	rs6546838	8	A/A	G/A	A/A	A
p.(Ser2112Arg)	rs6724782	8	T/T	A/T	T/T	T
p.(Arg2285Leu)	rs6546839	8	G/G	C/G	G/G	G
p.(Arg2827Ser)	rs2056486	10	G/G	G/G	G/G	G
p.(Asn2857Ser)	rs10193972	10	A/A	G/A	A/A	A
p.(Asn1787Asp) ^-^	rs45608038	8	A/G	A/G	G/G	G
p.(Tyr1714Ter) *	rs772136379	8	T/G	T/G	G/G	G

*: causal mutation. ^-^: rare variant linked to causal mutation.

## Data Availability

The data presented in this study are openly available in ClinVar.

## Supplementary Materials

The following are available online at https://www.mdpi.com/2073-4425/12/2/282/s1, Table S1. Pathogenic variants register in gnomAD and clinVar from the total alleles in our cohort. Table S2. Oligonucleotides for genomic amplification and sequencing of *ALMS1* described in Collin et al., 2002.
