# Supplementary material for: Prevalent ALMS1 Pathogenic Variants in Spanish Alström Patients
Source: Genes (Basel). 2021 Feb 16;12(2):282. doi: 10.3390/genes12020282 (PMC7920446; doi:10.3390/genes12020282)
Supplement: Supplementary file 1 [file genes-12-00282-s001.pdf]

Table S1. Pathogenic variants register in gnomAD and clinVar from the total alleles in our cohort.

	gnomAD variants	ClinVar variants
p.(Arg269Ter)	-	-
p.(Ser615Ter)	-	-
p.(Glu929Ter)	-	-
p.(Arg1417GlyfsTer55)	-	p.Arg1418fs
p.(Leu1424Ter)	-	-
p.(Glu1492Ter)	-	-
p.(Tyr1714Ter)	p.Tyr1713Ter	-
p.(His1808GlufsTer20)	-	-
p.(His2523ArgfsTer11)	-	-
p.(Val3596GlufsTer4)	p.Val3595GlufsTer4	p.Val3597fs
p.(Ser3872TyrfsTer19)	p.Ser3871TyrfsTer19	-

Table S2. Oligonucleotides for genomic amplification and sequencing of *ALMS1* described in Collin *et al*. 2002 [10].

Exon	Forward Primer	Reverse Primer
1	GCACTGCGCCTAAGCTG	CAGCCTCCACCCCCAAC
2	ATGTGAAAGGGCTTTATAAACTGG	TTTTTCCATTCTTCATAGCTAAATCA
3	CAGTTAATGACTTAGCATGTTTTCCT	TCCTTAACTCAAAAAGGGGAAAG
4	ACGTAAGTAAATAATCAATTTTCAGCA	TCTAAGCCCCACCTCAAAGT
5	TTTCAGTGACATATGTATTTTTGTGTT	TTCCCTTGGGAATTTTATTTTT
6	CTTCGTGTGTGGGAGCTGAG	CAATACTGAAAAAGGCCACGTT
7	TGGGCATTAATGAGTCTTTTTC	TTTTCACAAGGTATCCGTAAGTAGG
8	GCTTTTTAAAGGCTCAAAGCTG	TCTCTCTATGTGAGTAGGAAGTAGAGG
8	TGACCAGACAACTGGCATGT	GACTGTCTGCTAAGTCCTGTGG
8	TTCTTACTCACAAAGAGAAAAGCCTA	GGGCAGCCAATACAGAAACA
8	TTTCCCTGAAGAAGCTCTGAA	TGGCAAGGTCTGTTGGTAGA
8	TCACAAAGAGAGAAGCCTGGT	AGCTGGTGTGCCAGTTGTCT
8	TTCAGTTGCCTCTGAACCAG	TGTGGCAAGACCTGTTGGTA
8	CACACACAGAGAAGCCTGGT	AAAGGTCCTGCTGGTATGTCA
8	TCCATTGTTTCTGGACCTACTG	ATCTGGCAACTCTTGCTGGT
8	ACTGTAACTTCCTCTTTCTATTCACAT	TCTCAGTCTTCCGGTCACCT
8	AGCAGGAGTTGCCAGATGTT	CTGGTTTTCCAGTATTCACATCA
8	AAAGATTTCAGCTGTCCCTGA	CTGCATCCTGGATTTCTTCA
8	CTCAGGCTGATGACAGAGTTG	CCCAATGGTTCCACTACACC
8	GAGCAAAGTCAGTATGGCATTAGA	TGGCTAAGCTTCCTCAAAACA
9	TCTTCTGTGTTGCAATTGTTGA	TTCCATCACCCATTCTTTCA
10	TTGGACTACTTCAAATAAGAACCTG	GACGGCATTTGTGATGAAGA
10	ACCTGCTTTTGTGCCACCTA	CTTGGTCTGCCCATGCTAAT
10	CCAGTACCAGGGCAAATTGT	GGAAGGGGAAAATGGTGTTT
10	ACCTTCCGTCTCCCATTTCT	TCCTGTGCTACAGGTTTACTGG
10	GCTTCTAAAGCGAGGATGAA	CCCCCAAGAACCGATATCTA
11	TTCCTTGAAACCACTTTTGGA	GAAAGACACAACCACAAATTTCTAA
12	GAAGGCATTCCATATTTGTTCA	GCACTGGACTTTTGTCACTCC
13	TCATAGAATTGGTCTAAGAGGCAAA	AAGATTGGATAGTAATCTCATTTAGGA

14	ATGGGTTTGGGGTTTTGTTT	GAGCTGAAGACAGCAAGAAGAA
15	AACAAAGCCTTTCACATAATACG	CACTGACCCTCACATACACAC
16	GCAGGCAGTGAATTTTCTGAT	TTTTGGATAATCTCTAACTTGACTTTT
16	CCAGAATAAAGAGCCTCAGCA	TTTTTAAGCTCGCCTGTATTTTT
16	GCGGTTTAAAAGCCTAGAGAAA	TTTTCACCTGTGTGCAAAGC
17	TGAATTGGATTAGAAAGAGGACTTG	TCTTACATGTTTAAGAGCCATTTCA
18	TCCCACACAAAGGGATTGTA	ATCGCAGGGGACTTGAAAT
19	CTGGGTGGGGCTGTAAAAA	CCAAGTCACAGAGCCAGCTT
20	GCATATGGAGAGTAGATTGCATCA	TGGGCTGGCCTTTAGCAG
21	GGTAGGGGCACCAAGTCCTA	CAGAGCTCCCGACCACTTG
22	GATGAGCTCCTGGAGAGTGG	GGCAACGTGTTTTCTCCATT
23	GGCATCTGCCTCTGATGG	AAGGATTCTGCTTCTCTAGGTTCA
